# Development process of artificial intelligence based chatbot to support and promote mental wellbeing in sparsely populated areas of five European countries

**DOI:** 10.1192/j.eurpsy.2022.446

**Published:** 2022-09-01

**Authors:** L. Kuosmanen, A.-K. Vartiainen, H. Nieminen, C. Kostenius, R. Bond, M. Mulvenna, C. Potts, E. Ennis, M. Malcolm, A. Vakaloudis, B. Cahill, I. Dhanapala

**Affiliations:** 1 University of Eastern Finland, Department Of Nursing Science, Kuopio, Finland; 2 University of Eastern Finland, Department Of Health And Social Management, Kuopio, Finland; 3 Luleå University of Technology, Department Of Health, Education And Technology, Luleå, Sweden; 4 Ulster University, School Of Computing, Belfast, United Kingdom; 5 Ulster University, School Of Psychology, Londonderry, United Kingdom; 6 NHS, Western Isles, Western Isles, United Kingdom; 7 Munster technological University, Institute Of Technology, Cork, Ireland

**Keywords:** Promotion, wellbeing, chatbot, mental health

## Abstract

**Introduction:**

In many countries, people face problems regarding access to care, 24/7 support and evidence-based support. Digital interventions and services, such as chatbots, can be one option to tackle these challenges. There is a lack knowledge regarding how mental health chatbots are developed and how to ensure that there is collaboration between mental health and digital technology experts and users.

**Objectives:**

This presentation describes the phases of the development for the ChatPal mental health and wellbeing chatbot.

**Methods:**

Development process was conducted in five and with four different languages. First, using an electronic survey for mental health professionals (n =190) we screened how familiar they are with chatbots and how they evaluated their potential. Second, university students and staff, mental health professionals and service users (n=78) participated in workshops to design the chatbot content. Finally, the content and scripts of chatbot were written in multi-professional and multi-national collaboration.

**Results:**

ChatPal is based on the PERMAH model of positive psychology and on the idea that we all have mental health which needs boosting and support from time to time. ChatPal includes relevant mental health information, exercises, mood diaries and simple monitoring and self-care tools. Based on preliminary evaluations, the ChatPal chatbot offers an option to offer support in areas where other mental health services are lacking or are insufficient.

**Conclusions:**

ChatPal is already freely available in application stores and first scientific trials are have started. Preliminary results of 4-week and subsequent 12-week in-the-wild trials will be in place at the time of EPA 2022 conference.

**Disclosure:**

No significant relationships.

